# Bacterial taxonomic and functional profiles from Bohai Sea to northern Yellow Sea

**DOI:** 10.3389/fmicb.2023.1139950

**Published:** 2023-02-23

**Authors:** Tianyi Niu, Yongqian Xu, Jinni Chen, Liangyun Qin, Zhicong Li, Yating Yang, Jiayuan Liang

**Affiliations:** ^1^School of Marine Sciences, Guangxi University, Nanning, China; ^2^Coral Reef Research Center of China, Guangxi University, Nanning, China; ^3^Guangxi Laboratory on the Study of Coral Reefs in the South China Sea, Nanning, China

**Keywords:** microbial distribution patterns, marine ecosystem, bacterial community structure, biogeography, co-occurrence patterns, functional variation

## Abstract

Microbial distribution patterns are the result of a combination of biotic and abiotic factors, which are the core issues in microbial ecology research. To better understand the biogeographic pattern of bacteria in water environments from the Bohai Sea to the northern Yellow Sea, the effects of environmental factors, and spatial distance on the structure of bacterial communities in marine water were investigated using high-throughput sequencing technology based on 16S rRNA genes. The results showed that Proteobacteria, Bacteroidetes, Actinobacteri, Desulfobacterota, and Bdellovibrionota were the dominant phyla in the study area. A clear spatial pattern in the bacterial community was observed, and environmental factors, including salinity, nutrient concentration, carbon content, total phosphorus, dissolved oxygen, and seawater turbidity emerged as the central environmental factors regulating the variation in bacterial communities. In addition, the study provides direct evidence of the existence of dispersal limitation in this strongly connected marine ecological system. Therefore, these results revealed that the variation in bacterial community characteristics was attributed to environmental selection, accompanied by the regulation of stochastic diffusion. The network analysis demonstrated a nonrandom co-occurrence pattern in the microbial communities with distinct spatial distribution characteristics. It is implied that the biogeography patterns of bacterial community may also be associated with the characteristics of co-occurrence characterize among bacterial species. Furthermore, the PICRUSt analysis indicated a clear spatial distribution of functional characteristics in bacterial communities. This functional variation was significantly modulated by the environmental characteristics of seawater but uncoupled from the taxonomic characteristics of bacterial communities (e.g., diversity characteristics, community structure, and co-occurrence relationships). Together, this findings represent a significant advance in linking seawater to the mechanisms underlying bacterial biogeographic patterns and community assembly, co-occurrence patterns, and ecological functions, providing new insights for identifying the microbial ecology as well as the biogeochemical cycle in the marine environment.

## Introduction

Microorganisms play a pivotal role in marine ecosystems and participate in various geochemical processes in marine waters, which are essential for maintaining the stability of marine ecosystems (Pajares and Ramos, 2019; Fu et al., 2020). Microbial biogeography is an exciting topic in microbial ecology, and it is essential to explore the mechanisms regulating the diversity and spatial and temporal distribution patterns of microbial communities (Nemergut et al., 2013). Marine microbial communities are more susceptible to environmental changes than other ecosystems because of the tight environmental connectivity and environmental heterogeneity of marine water environments. Numerous studies have shown that the spatiotemporal dynamics of marine microbial communities depend on the characteristics of the marine environment (e.g., salinity, temperature, and ammonia concentration), and their heterogeneity shapes the spatial and temporal distribution patterns in marine microbial community structures (Gilbert et al., 2012; Ward et al., 2017; Liu et al., 2019). Furthermore, the geographical distance also exerting an effect on the distribution pattern of marine microorganisms (Wu et al., 2018). At larger spatial scales, the geographical distribution of microorganisms responds to changes in environmental factors combined with spatial distance, as they represent deterministic and stochastic processes of microbial community construction, respectively, and jointly determine the geographical distribution patterns of microorganisms (Zhou and Ning, 2017).

The advent of high-throughput sequencing technologies has allowed for the thorough description of diversity, composition structure and interrelationships of microbial communities in natural environments, which has enhanced our understanding of microbial ecological relationships and ecological functions in specific environments (Barberán et al., 2012; Tinta et al., 2015; Jiao et al., 2016). Microbial taxa experience complex interrelationships, such as competition, mutualistic symbiosis, and antagonism, which underpin community stability and function (Faust and Raes, 2012). Such network interactions also dramatically affect the mechanisms of microbial community composition structures as well as ecological functions (Jiao et al., 2016, 2020), followed by maintaining the stability of microbial diversity and function. However, due to limited techniques, elucidating microbial interrelationships in complex natural environments has remained challenging. Species abundance-based correlation network analysis plays a critical role in understanding microbial interrelationships within complex microbial communities in the environment (Zhang et al., 2014; Zhou et al., 2018). Moreover, the co-occurrence patterns of microbial communities are also vulnerable to community assembly. Thus, the co-occurrence patterns of microbial communities reflect, to some extent, the contribution of deterministic and stochastic processes to microbial community composition (Kraft et al., 2015; Jiao et al., 2020).

The Bohai Sea, located in northeastern China, has a mean depth of 18 m and is an almost-enclosed inland sea. In recent decades, the marine environment in the Bohai Sea region has been subject to severe seawater eutrophication and seasonal hypoxia due to the continuous influence of industrial production and sewage discharge from surrounding cities (Zhai et al., 2012; Qiao et al., 2017). The Yellow Sea, in a vast terrain, is adjacent to the Bohai Sea with an average depth of 44 m. Ocean currents and monsoons trigger a close material circulation between the two waters. In addition, the runoff of the upper river (the second largest river in China, the Yellow River) induces accumulated organic matter, ammonia and pollutants from terrestrial origin to enter the adjacent northern Yellow Sea *via* the Bohai Sea, which results in a significant seawater environmental gradient (e.g., salinity, temperature, and nutrient content) in this region (Dang et al., 2013; Chen et al., 2019). Therefore, this region is considered an ideal place for studying the ecological and functional characteristics of marine microorganisms. In recent years, numerous studies have focused on the spatial and temporal dynamics and regulatory mechanisms of microbial community structure and diversity in the Bohai Sea, as well as the Yellow Sea (Yu et al., 2018; Chen et al., 2020; Li et al., 2022; Wang et al., 2022). Identifying the mechanisms behind seawater bacterial community construction, relationships, and potential geochemical functions in this region is essential for better understanding the ecological characteristics and function of bacterial communities.

In this study, we investigated the distribution pattern of bacterial communities in seawater from the Bohai Sea to the northern Yellow Sea on a spatial scale and explored the effects of environmental factors (as a niche-based process) and dispersal limitation (as neutral-based processes) on bacterial community structure variation. Furthermore, the assembly processes and co-occurrence patterns of the bacterial community were assessed to explore the mechanism of bacterial communities. Then, the sequence data were further employed in PICRUSt (phylogenetic investigation of communities by reconstruction of unobserved states) analysis to examine the ecological functions of bacterial communities in this region. Finally, this study aims to uncover the connections between functional profiles and biodiversity in the bacterial community.

## Materials and methods

### Sample collection and sediment physicochemical indicators

**Figure 1:**
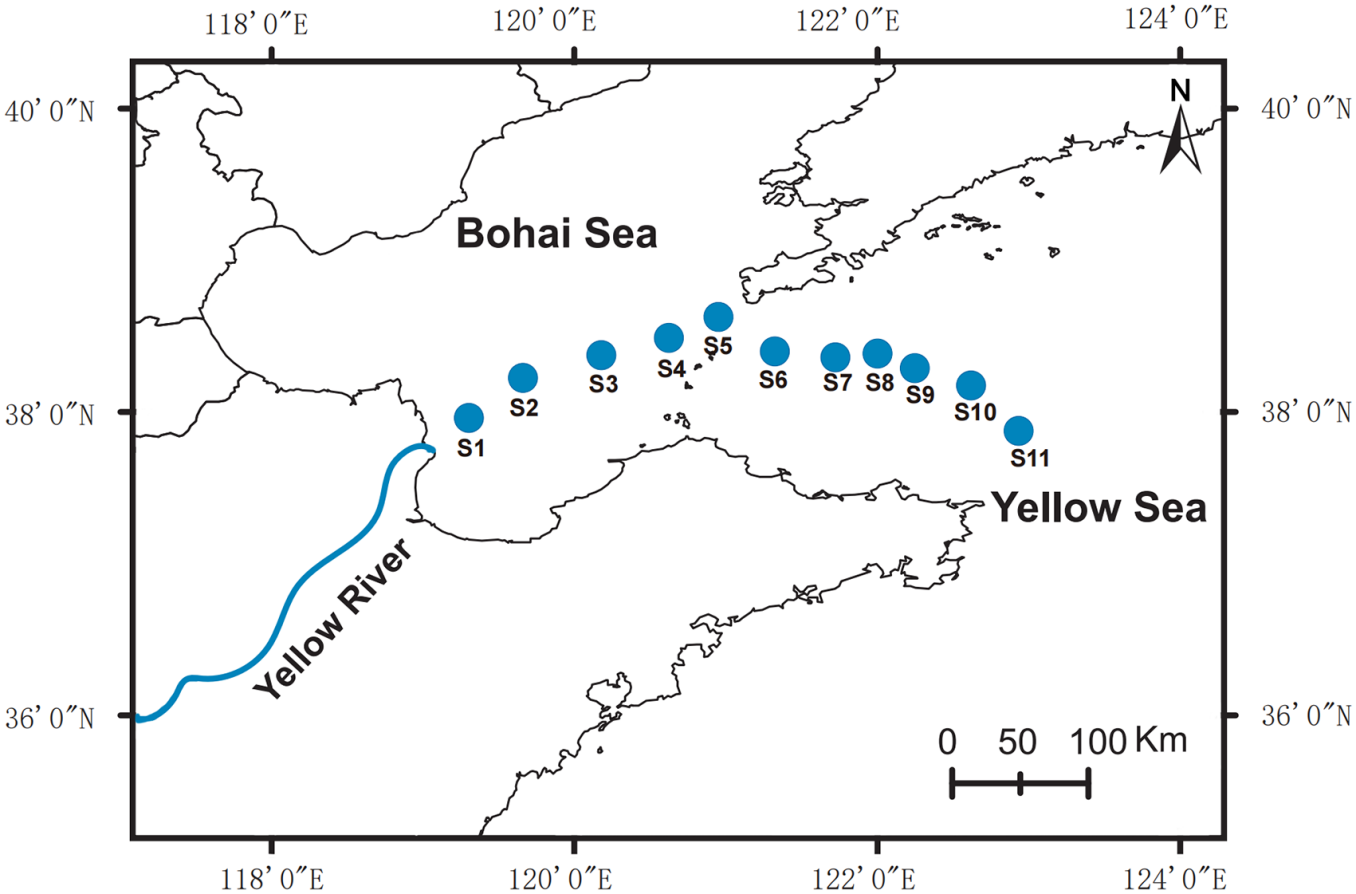
Location of sampling sites (S1–S11) in this study.

The sampling sites were located at the edge of the Yellow River Estuary to the northern Yellow Sea (Figure 1). At each sampling point, 2 liters of seawater was collected from the surface layer (0–1 m) and the bottom layer (2–3 m above sediment), respectively. For further DNA extraction, each sample was coarse filtered through 0.22 mm pore-size white polycarbonate filters. The cell pellets on the polycarbonate membranes were immediately sealed in sterile 50 mL centrifuge tubes and stored at –80°C.

As listed in Supplementary Table 1, the environmental parameters including salinity, temperature, and depth were measured *in situ* using a CTD sensor (Sea-Bird Electronics Inc., Bellevue, WA, United States). Solution pH and dissolved oxygen (DO) were detected on a boat with a probe (Hydrolab MS5; HACH, Loveland, CO, United States). Concentrations of ammonium (NH_4_^+^), nitrite (NO_2_^−^), and nitrate (NO_3_^−^) were measured by a nutrient flow analyzer (Seal, Norderstedt, Germany). The ascorbic acid-molybdate blue method was adopted for assaying total phosphorus (TP), and the Vario EL CN Elemental Analyzer (Elementar, Germany) was used to measure the total organic carbon (TOC). Turbidity was recorded in terms of nephelometric turbidity units (NTU), which was then performed for the calculation of suspended sediment concentrations (SSC, in g L^−1^) according to the method described by Chanson et al. (2008). All physiochemical parameters were measured in triplicate.

### Bacterial 16S rRNA high-throughput sequencing method

The total DNA was extracted from the filtered membranes using the PowerWater DNA isolation kits (MP BIO, Santa Ana, CA, United States) as directed by the manufacturer’s instructions. The quality of the DNA extracts was determined by agarose gel electrophoresis. The verified DNA products were subjected to concentration measurement by a NanoDrop 2000c spectrophotometer (Thermo Fisher, Waltham, MA, United States).

PCR amplification of the V3-V4 variable region of the bacterial 16S rRNA gene was performed using primers 338F/806R. The PCR system was implemented in 50 μL, containing DNA template 50–100 ng, primers 20 pmol/L each, 4 × dNTP (2.5 mmol/L each) 5 μL, MgCl_2_ 2.5 mmol/L, 10 × PCR buffer 5 μL, BSA 300 ng/mL, Taqase (5 U) 0.2 μL, and ddH_2_O to 50 μL. The amplification reaction was performed under the following conditions: 94°C for 3 min; 94°C for 30 s, 50°C for 30 s, 72°C for 30 s, 35 cycles, and 72°C extensions for 10 min. All samples were amplified in triplicate, and amplification without template was used as a control. PCR products in 5 μL were subjected to 2% agarose gel detection and cut-gel recovery with a gel recovery kit (Qiagen, Hilden, Germany). PCR product concentrations were determined by the Quant-iT PicoGreen dsDNA (Life Technologies, Merelbeke, Belgium) kit. Sequencing was performed using the Illumina MiSeq (250-bp) platform. The obtained sequences were denoised using Denoiser V0.91 software and chimeras were removed using USEARCH software. Sequences were clustered into operable taxonomic units (OTUs) with a 97% identity threshold with uclust using Mothur software (Caporaso et al., 2011). One representative sequence from each OTU was selected for annotation of the classification units using the RDP classifier at an 80% confidence threshold based on the SILVA database. Prior to performing the data analysis, a minimum number of sequences (31,874) was drawn flat for all samples to eliminate the effect of different sequence sizes of the samples.

### Data processing and statistical analysis

The bacterial community α-diversity indices (including Chao1 index, Shannon index, Simpson index, etc.) were calculated by Mothur software, Hellinger-transformed data were used to generate a Bray-Curtis distance matrix by the “vegan” package in R environment, and nonmetric multidimensional scaling (NMDS) was used to evaluate the taxonomic and functional differences in the microbial community. One-way analysis of variance (ANOVA) were conducted to compare spatial variation of bacterial community structure on phylum level using Statistical Package of Social Sciences software (SPSS, version 22.0). The differences between groups were tested using a permutational analysis of variance fixed model (PERMANOVA). Redundancy analysis (RDA) was used to identify the environmental indicators that formed microbial structure. The Mantel test was used to test the correlation between Bray-Curtis distance matrices. The pairwise geographic distances between samples were calculated using the “geosphere” package in R, which also compares the Bray-Curtis differences using the “ggplot2” package. The Spearman correlation between Bray-Curtis differences and geographic distances was calculated. The principal coordinate proximity method (PCNM) was used to extract spatial variables. The explanations behind colony variation were investigated from environmental factors and spatial variables in the RDA to distinguish their relative weights using variance decomposition analysis (VPA). Welch’s test (confidence interval method) in STAMP software was used to identify differences in the relative abundance of taxonomic and functional microbiota under different spatiotemporal groupings. Null model analysis was performed using the framework of Stegen et al. (2013) to classify community assembly as potential drivers by deterministic processes (including homogeneous and heterogeneous selection), dispersal limitation, homogeneous dispersal and other (undominated). The βNTI index (β-nearest taxon index), and the Raup Crick index (RC_bray_) were confirmed to measure both phylogenetic turnover and taxonomic turnover using a null model (Liu et al., 2020).

Co-occurrence network were used to explore the interrelationships among microorganisms. The top 100 OTUs with relative abundance were selected for Spearman correlation analysis. OTUs with correlation coefficients greater than 0.6 and significant *p* values less than 0.01 were screened out to construct correlation networks (Barberán et al., 2012). Each node in the network graph represents an OTU, and each edge represents a significantly potent correlation between nodes. The topological parameters of each network graph were calculated, including the average path length, network diameter, clustering coefficient, modularity, average degree, and network density. Each node of the network graph is colored according to modularity attributes and annotated with OTUs. The networks are visualized in Gephi software. Statistical analysis was carried out in R environment (v3.5.1^1^) for all processing unless otherwise specified.

The potential functions of microbial communities were predicted based on the 16S rRNA gene phylogenetic characteristics by using PICRUSt2.^2^ Predicted gene class abundances were analyzed at KEGG (Kyoto Encyclopedia of Genes and Genomes) and defined in KEGG level 2.

## Results

### Bacterial alpha diversity and its taxonomic composition

**Figure 2:**
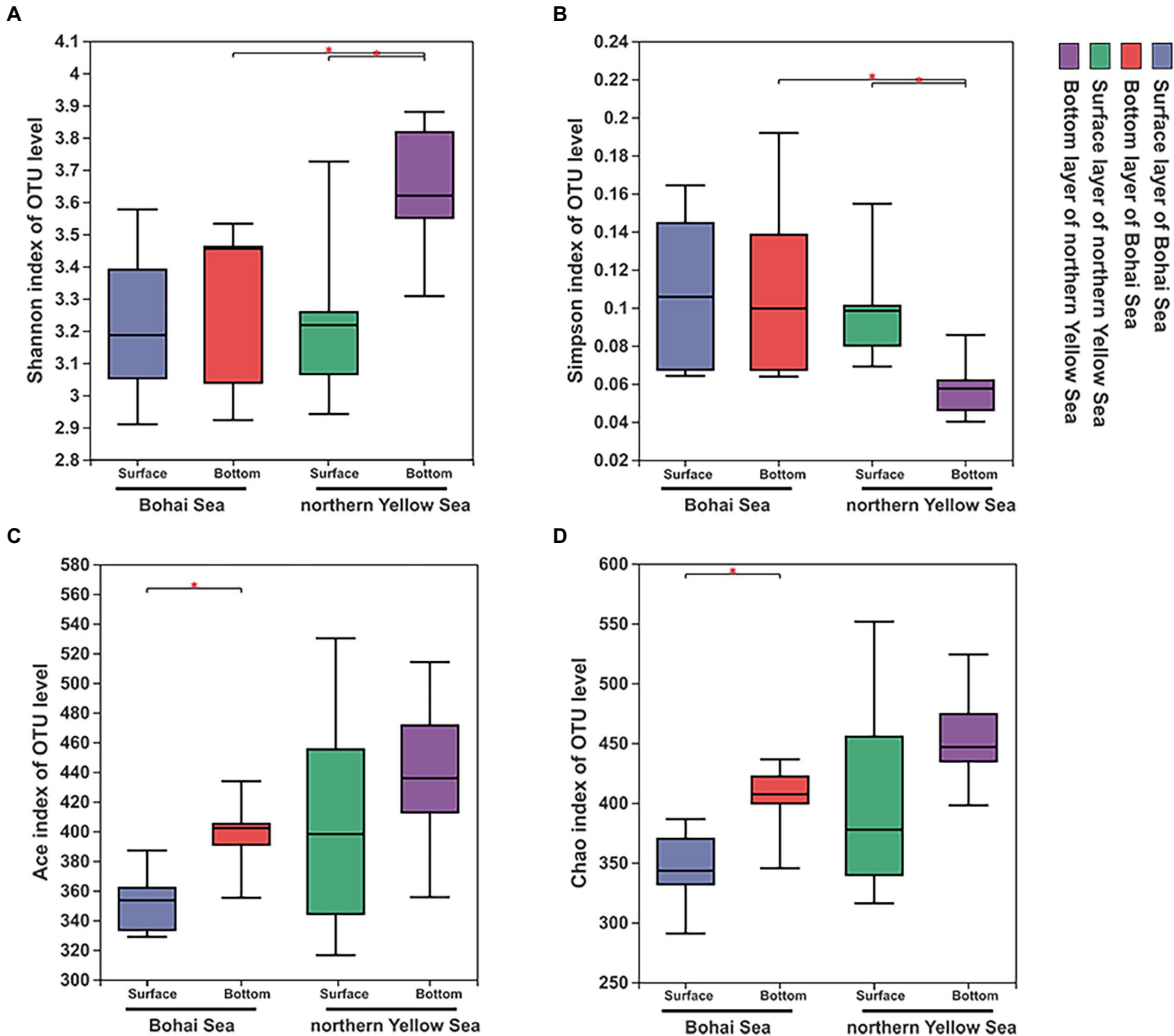
Diversity characteristics, including the Shannon **(A)**, Simpson **(B)**, Ace **(C)**, and Chao 1 **(D)** indices, of bacterial communities along the Bohai Sea to northern Yellow Sea. Significant differences between water layers in each sampling area are marked by stars (**p < 0.01; *p < 0.05).

After quality filtering, chimera deleting and sequence assembly, a total of 1,918,952 reads were obtained after 16S rRNA gene sequencing, and 1,490,046 high-quality sequences were finally retrieved by double-end splicing and filtering. Based on the minimum number of sequences (31,874), 942 OTUs were finally obtained with 97% similarity. Rarefaction curve results showed that the observed number of OTUs leveled off with increasing sequencing depth, indicating that the sequencing depth of this experiment could cover most of the bacterial species (Supplementary Figure 1). In the vertical direction, the samples in the bottom illustrated more abundant OTUs compared to those in the surface samples. Simultaneously, the number of OTUs in the northern Yellow Sea samples was markedly expanded compared with that in the Bohai Sea samples. The overall samples had Chao I and ACE indices ranging from 290.833 to 551.592 and 316.516 to 530.089, respectively. The total Shannon and Simpson indices ranged from 2.910 to 3.880 and 0.0401 to 0.192, respectively (Supplementary Table 2). The richness index and diversity index of the bacterial community increased with increasing water depth, and the northern Yellow Sea had significantly higher diversity characteristics in the bottom layers than in the surface layer (Figures 2A,B). The richness indices including the Ace and Chao indices of the bacterial community were significantly higher in the bottom samples from the Bohai Sea (Figures 2C,D). The horizontal direction also experienced a trend of increasing diversity (Figures 2A,B) and richness (Figures 2C,D) of the bacterial community from offshore to deep water.

The total bacteria revealed by the 16S rRNA gene at the phylum level showed that Proteobacteria was observed with a dominant abundance among all samples, occupying 77.01% of the community. The following phyla were detected: Bacteroidetes (18.28%), Actinobacteria (1.25%), Desulfobacterota (1.14%) and Bdellovibrionota (1.04%; Supplementary Figure 2A). Significantly higher proportions of Desulfobacterota were detected in bottom-water layers (one-way ANOVA, *p* = 0.015), which presented the highest abundances at site S9 (Supplementary Figure 2B). By comparison, the relative abundances of dominant bacterial phyla horizontally exhibited significant variations from the Bohai Sea to the Yellow Sea, indicating significantly higher proportions of Bacteroidetes and Actinobacteria in the Bohai Sea (one-way ANOVA, *p* = 0.047).

### Bacterial community β-diversity characteristics and influencing factors

**Figure 3:**
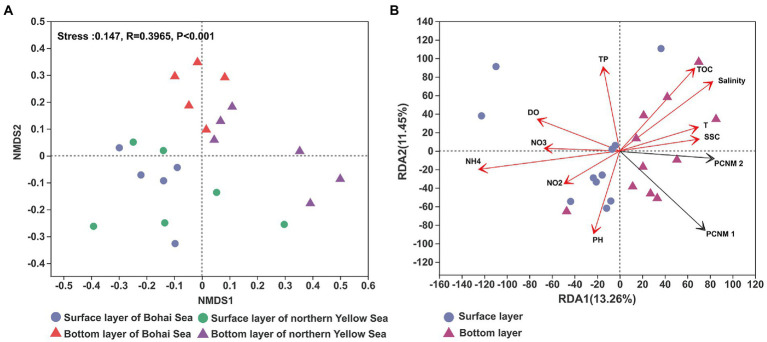
The NMDS of the bacterial community revealed by the 16S rRNA gene sequences **(A)** and RDA of the distribution of bacterial communities with environmental factors **(B)**. T, TOC, DO, SSC, TP, NO3, NO2, and NH4 represent temperature, total organic carbon, dissolved oxygen, suspended sediment concentration, total phosphorus, nitrate, nitrite and ammonium, respectively.

The community structure of bacteria has been reported to be different between water layers. The NMDS result showed that surface seawater presented a considerable difference in bacterial community structure compared with bottom seawater (Figure 3A). From the Bohai Sea to the Yellow Sea, the structure of the bacterial community in the surface seawater was generally conserved, while the bacterial community in the bottom seawater demonstrated an obvious spatial distribution. The bacterial community from the Bohai Sea and the bacterial community from the Yellow Sea clustered together. RDA results revealed that the distribution pattern of bacterial communities was related to the spatial heterogeneity of the seawater environment. A variety of environmental factors served as the leading indicator that regulated the changes in bacterial communities, including salinity, nutrient variables (NH_4_^+^ and NO_3_–), total organic carbon (TOC), total phosphorus (TP), dissolved oxygen (DO), and seawater suspended sediment concentrations (SSC) (Figure 3B). Furthermore, the variance decomposition analysis (VPA) based on RDA results showed that environmental factors accounted for 16.71% of the microbial community variables, whereas 75.29% of the variables remained unexplained compared to spatial factors (Residuals = 75.29%; Supplementary Figure 3).

### Bacterial community co-occurrence patterns

**Figure 4:**
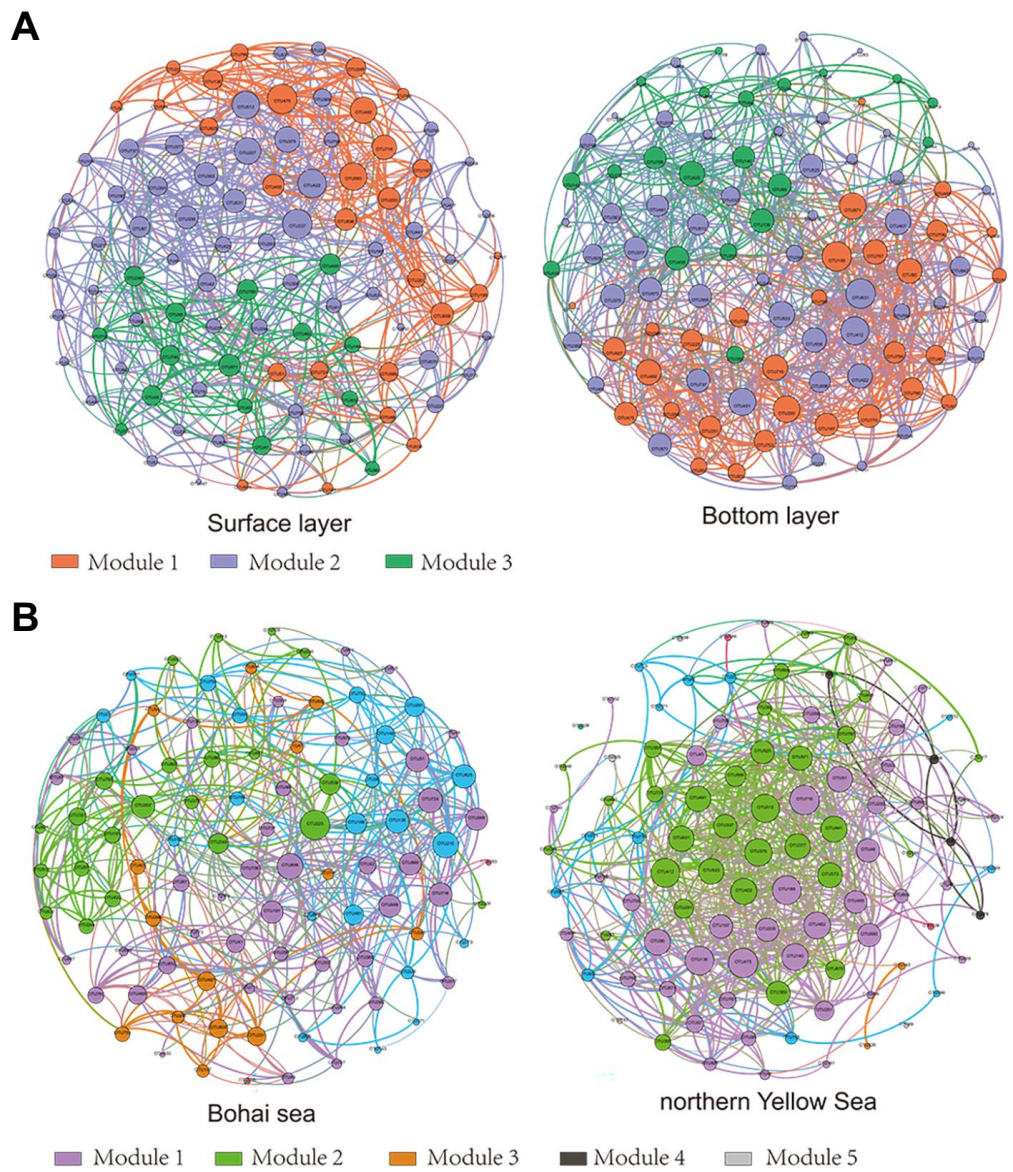
Network of bacterial OTUs based on correlation analysis between the surface and bottom layers **(A)** and between the Bohai and northern Yellow Seas **(B)**. A connection stands for a strong (Spearman’s *r* > 0.6) and significant (*p* < 0.01) correlation. Co-occurring network colored by modularity class. The size of each node is proportional to the relative abundance; the thickness of each connection between two nodes (edge) is proportional to the value of Spearman’s correlation coefficients.

The results of the OTUs correlation network based on the top 100 abundances showed significant clustering of bacterial communities in different seas and water layers (modularity index; MD > 0.4). Such observations suggested that a co-occurrence pattern of bacterial communities was composed of highly connected patterns and formed a “small world” topology. Bacterial communities existed in 669 and 907 connections (edges) in surface and bottom seawater samples (Figure 4A), respectively, and 481 and 731 connections (edges) in the Bohai and northern Yellow Sea waters (Figure 4B), respectively. This indicates that there was a closer relationship among bacterial communities in bottom seawater, as well as in northern Yellow Sea water. The microbial co-occurrence network results showed that most of the links experienced positive correlations (53.25–62.78%), higher than negative correlations (42.00–46.75%), indicating that species tended to co-occur rather than coexclude in all the microbial networks.

Additionally, the network topology results showed that the bacterial co-occurrence patterns presented remarkable discrepancies in different water layers as well as in different seas. Compared with the surface layer, a tighter co-occurrence pattern occurred in the bottom seawater of the bacterial communities, and their network topological parameters, including modularity index (MD), graph density (GD), average degree (AD), network diameter (ND), and clustering coefficient (CC), were larger than those in the surface layer bacterial communities. At the horizontal scale, the bacterial communities in the northern Yellow Sea had a closer co-occurrence pattern than those in the Bohai Sea (Table 1).

**Table 1:** Topological properties of the bacterial co-occurrence network.

	Nodes	Edges	Average Path Length (APL)	Modularity index (MD)	Graph Density (GD)	Average Degree (AD)	Network Diameter (ND)	Clustering Coefficient (CC)
Surface layer	100	669	2.303	1.530	0.135	13.38	4	0.443
Bottom layer	100	907	2.161	8.424	0.183	18.14	5	0.531
Bohai Sea	100	481	2.640	3.227	0.097	9.62	5	0.463
northern Yellow Sea	100	731	2.517	2.022	0.148	14.62	7	0.519

### Bacterial community assembly process

**Figure 5:**
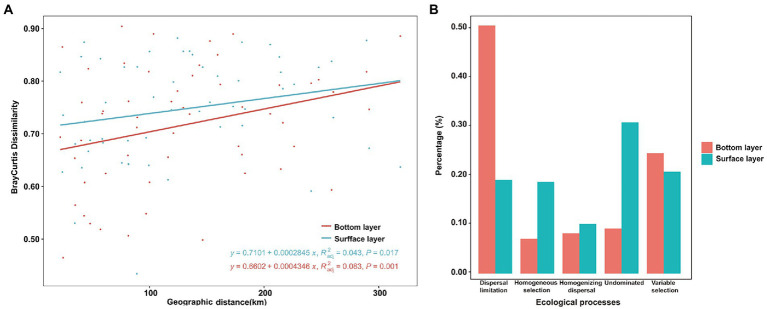
Bray–Curtis dissimilarity patterns of the bacterial community **(A)** and null model analysis revealing the assembly processes **(B)** of the bacterial community in the bottom and surface layers.

A more significant positive distance decay relationship of bacterial similarity (*R* = 0.083, *p* < 0.001) was found in the bottom bacterial community (Figure 5A) compared to the surface bacterial community (*R* = 0.043, *p* = 0.017). To explore the mechanisms underpinning the observed distribution pattern and co-occurrence patterns, the relative roles of niche and neutral processes in community assembly were analyzed. In surface-layer water, variable selection, as a part of the deterministic process, was responsible for 20.86% of the community variation across all samples, along with dispersal limitation in stochastic processes, accounting for 19.21% of the overall variation. Moreover, 30.96% of the undominated compositional turnover of bacteria was observed in the surface layer (Figure 5B). However, bacterial community variation was more susceptible to dispersal limitation in stochastic processes, followed by variable selection in bottom-layer water (Figure 5B).

### Potential functional characteristics

**Figure 6:**
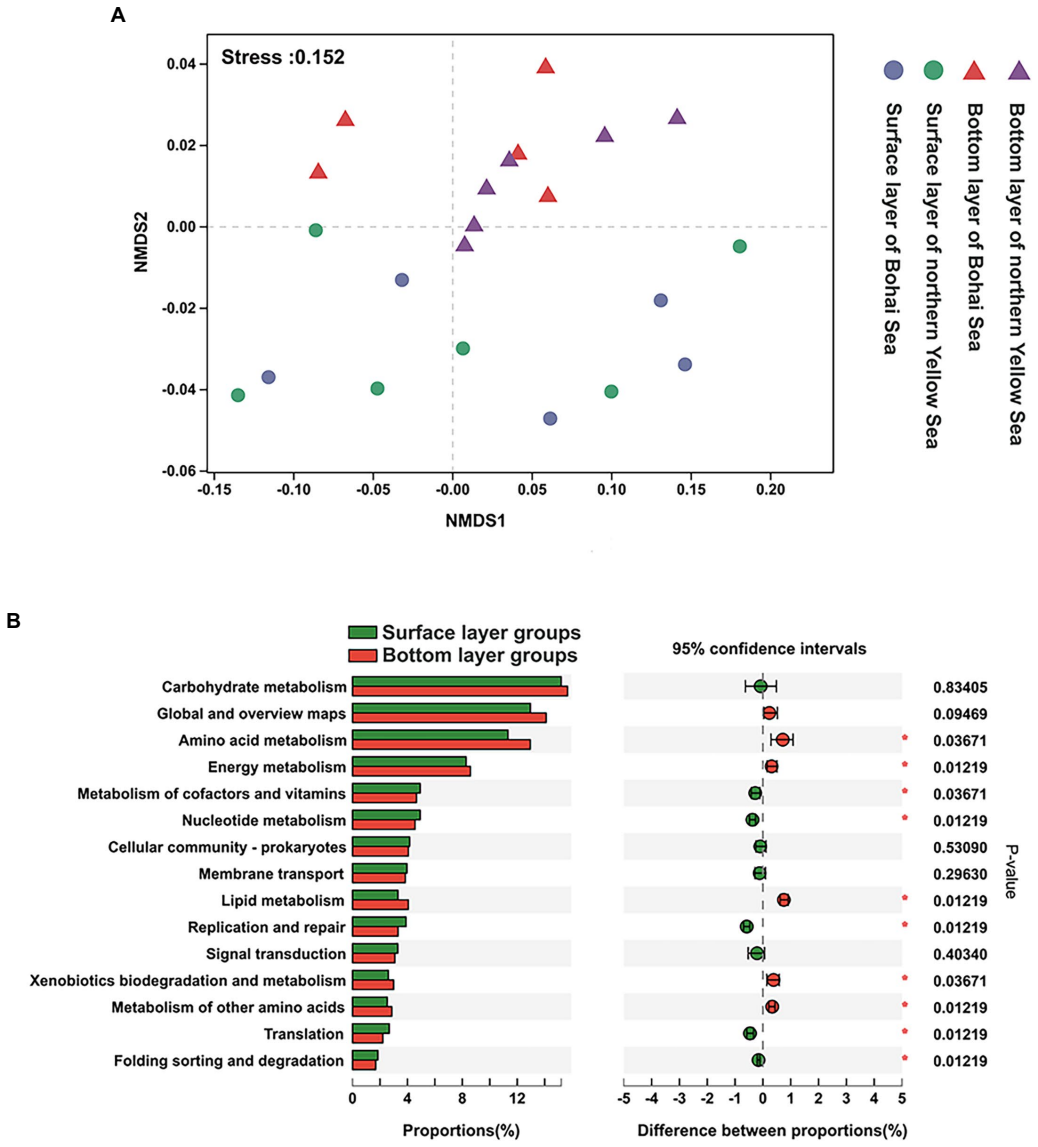
**(A)** NMDS of the predicted function of KEGG pathway 2. **(B)** Statistical comparison of the relative abundance of dominant functional characteristics in the bottom and surface layers. * considered significant difference, *p* < 0.05.

The predicted functional characteristics formed a similar pattern as the bacterial community structure, which reached statistical significance between the surface bacterial community and those of the underlying community, albeit not significant in the horizontal direction (Figure 6A).

At KEGG pathway level 2, the global and overview maps, amino acid metabolism, carbohydrate metabolism, energy metabolism, metabolism of cofactors and vitamins, and membrane transport exhibited high abundance, which dominated at 64.59% in all functional abundance of the bacterial community (Supplementary Figure 4). It was also found that repair was always significantly highly represented in bottom-layer water (Figure 6B).

The CCA results showed that environmental factors, including salinity, nutrient concentration (NH_4_+ and NO_3_–), TOC, TP, DO, SSC, and bacterial community diversity (Simpson and Chao 1), were related to the functional characteristics of the Bohai to northern Yellow Sea. These results revealed that the bacterial diversity characteristics were significantly correlated with community function, highlighting the indispensable role of bacterial community diversity in its functional characteristics (Table 2).

**Table 2:** Correlation coefficient and significance test value of each factor on functional distribution pattern in RDA.

		RDA1	RDA2	*R* ^2^	*p* values
Environmental factors	Salinity	−0.46727	−0.88411	0.888321	**0.001**
	PH	0.994072	−0.10872	0.032075	0.823
	NH_4_^+^	−0.40952	−0.9123	0.797284	**0.001**
	SSC	−0.24171	−0.97035	0.369727	0.058
	NO_2_^−^	−0.33281	0.942993	0.229737	0.133
	TOC	−0.67507	−0.73775	0.323223	0.051
	NO_3_^−^	−0.57798	−0.81605	0.525633	**0.008**
	TP	−0.68787	−0.72584	0.267233	0.145
	Temperature	−0.59877	−0.80092	0.075713	0.624
Spatial factors	PCNM1	0.709591	0.704614	0.131198	0.447
	PCNM2	0.376416	0.926451	0.031207	0.835
Diversity factors	Shannon	−0.04533	−0.99897	0.043826	0.088
	Simpson	0.848706	0.528865	0.375795	0.057
	Chao 1	0.931655	0.363345	0.545633	**0.01**

Bold indicates the significant impact on functional profiles.

## Discussion

Bacterial communities emerge as the main taxa that mediate the material cycle and energy flow in marine waters, engaging in biogeochemical cycling to maintain marine ecosystem stability (Voss et al., 2013). In the marine environment, frequent natural events and increasing human activity dramatically lead to microbial community dynamics, which elicit an imbalance in biogeochemical cycles and alter ecosystem functioning (Hutchins and Fu, 2017).

This study found that the water layers were involved in the community diversity and composition of bacterial differentiation, which were significantly affected by environmental changes. The dominant groups were Proteobacteria and Bacteroidetes. This pattern presents a similarity to the patterns of other marine environments around the world (Liu et al., 2018; Wang et al., 2019), which may be explained by the presence of large amounts of foreign organic and inorganic compounds in this region (He et al., 2017). Studies have shown that Bacteroidetes play a pivotal role in predicting the early risk of marine red tides and the microbial degradation of marine polymers, promoting the healthy development of marine ecosystems (Teeling et al., 2012). Due to its special location, the Bohai Sea area is particularly disturbed by estuarine export and human activities; thus, the pooling of nutrients such as carbon, nitrogen, and phosphorus results in a higher abundance of Proteobacteria and Bacteroidetes in this area and complicates the diversity and abundance of microorganisms from the Bohai Sea to neighboring seas.

In accordance with the previous description (Needham et al., 2017; Liu et al., 2019; Reji et al., 2019), the results of this study showed that heterogeneity in environmental characteristics (e.g., salinity, temperature, pH characteristics) and resource abundance (e.g., nutrient concentrations) of marine waters drove changes in bacterial diversity and community structure both vertically and horizontally. Nutrients remarkably affect the composition and growth of bacteria in the environment, including nitrogen, carbon, and iron in the bioavailable form, which may be fully utilized by microorganisms and closely related to bacterial diversity (Soares et al., 2017). From the Bohai Sea to the northern Yellow Sea, the reason behind the high diversity and richness in the bacterial community may be explained by the presence of high nutrient concentrations in the bottom water column. At the horizontal scale, bacterial diversity was more vulnerable to salinity changes. The effects of environmental factors on bacterial communities vary from sea to sea and from water layer to water layer due to differences in topography, current velocity, degree of sediment suspension, and microbial sources. For example, in the surface layer of seawater, nutrient concentration was the dominant environmental factor affecting the spatial variation in bacterial communities, while the variation in bacterial communities in the bottom layer of seawater was critically attributed to salinity and TOC concentrations. In conclusion, the variation in microbial communities in terms of components implies that variations in environmental factors in different marine areas as well as in different water layers sorted the relative abundance of bacterial communities. A deterministic process (environmental filtering) assisted in shaping the bacterial community from the Bohai Sea to the northern Yellow Sea.

Microbial ecology studies have consistently considered abiotic selection as a niche-based (deterministic) process (also known as habitat filters) and ecological drift and dispersal limitation as neutral-based (stochastic) processes. This study found that the decline in bacterial similarity as the geographic distance increases from the Bohai Sea to the northern Yellow Sea. This implied that the dispersal limitations due to spatial distance may affect the biogeographic distribution of the bacterial community from the Bohai Sea to the northern Yellow Sea. Unlike terrestrial soil or sediment environments (Jiao et al., 2019; Wu and Huang, 2019), our VPA results showed that the environmental factor or spatial distance explained a relatively low community variation, and the pure environmental factor have a more important role compared with the pure spatial factors. This may be attributed to the highly connective ecological system of the seawater environment, and therefore, dispersal limitation due to geographical distance fails to elicit an effect on the turnover of the bacterial community structure.

The spatial and temporal variability of microbial communities was determined by the interaction of deterministic and stochastic processes (Chave, 2004). However, the relative importance of the two varies across spatial and temporal scales and is influenced by the degree of environmental variability and the sensitivity of microorganisms to environmental change. In general, excessive environmental variation prevents dispersal beyond the threshold a microbe can endure (Wang et al., 2013), leading to the predominance of determinism. Thus, the mechanisms underlying microbial community assembly would change with the magnitude of environmental heterogeneity (Dini-Andreote et al., 2015). The results of the null model in this study showed that stochastic processes have a greater effect on the assembly of bacterial communities than deterministic processes. This is not in agreement with the VPA results in this study, which possibility because that the VPA could incorrectly predicting the effects of environmental and spatial factors on community variation (Smith and Lundholm, 2010; Chen et al., 2019). Another explanation is that the physiochemical factors analyzed to represent environmental impact exhibit low relevance with community variations (Zhang et al., 2018; Liu et al., 2020). Bacterial communities often witness stochastic growth, mortality, migration and extinction events that also affect the spatial variability of bacterial community structure. This large unexplained community variation and high autocorrelation between spatial and environmental factors in this study may explain the disparity between VPA and the null model.

In the marine environment, marine microbes might have evolved strong adaptation capabilities to environmental changes; subsequently, spatial connectivity and seawater movement facilitate the homogenization of environmental conditions. An ecosystem with relatively uniform environments allows stochasticity processes to dominate the community assembly (Wu and Huang, 2019). Thus, deterministic processes may occupy the principal position on community variability in highly circulating as well as dramatically changing environments in the marine environment. Indeed, these results imply that spatial factors (stochasticity) were more important in the bottom bacterial community than in surface sea water.

Marine microbes are highly diverse and encompass taxonomically and functionally different lineages. Complex interactions occur among microbial taxa, which underpin community stability and functioning. The results in this study described a closer interaction between the bottom seawater bacterial communities, which may be the consequence of the stronger heterogeneity in the surface seawater environment, making the turnover rate of the surface seawater bacterial communities faster.

Additionally, strong river discharges contributed to the occurrence of the Yellow Sea Cold Water Mass (Zhang et al., 2008). These events may aggravate the separation of different water layers, thus resulting in an overall more scattered co-occurrence pattern in surface sea water. However, this study reported that the distance factor was taken as a prime trigger in bottom seawater of the bacterial community than in the surface layer, which may induce the co-occurrence pattern of the bacterial community in bottom seawater to be more dispersed. It may be speculated that the bacterial community in bottom seawater would show a more discrete co-occurrence pattern under the wider spatial scale of the survey area, or might been explained that the dynamics of microbial community structure changes and co-occurrence patterns are inconsistent and not necessarily correlated (Liu et al., 2020). However, it is still worth noting that the co-occurrence patterns obtained from topological results based on species correlation features do not reflect the co-occurrence patterns among the species shown (Ma et al., 2016).

A group of densely connected nodes with weak correlations to other nodes forms a module. Modular analysis provided favorable responses, as it simplified the processes of identifying keystone taxa and/or exploring the effect of environmental factors on microbe interactions. The results of the co-occurrence pattern analysis showed that the bottom seawater bacterial communities had more obvious modularity than surface seawater, with modularity coefficients of 8.424 and 1.530, respectively. The same module may confer similar functional characteristics to bacterial communities (Jiao et al., 2016). Therefore, the modularity characteristics of the co-occurrence patterns of marine bacterial communities from the Bohai Sea to the northern Yellow Sea reflect the differences in the ecological functions of bacterial communities in different water layers as well as sea areas. The strong gradients in nearshore to offshore microbial community composition, assembly processes and co-occurrence patterns raise the question of whether functional differences exist across these different transects.

The results of the study showed that there was clear stratification in the functional characteristics of the seawater bacterial community. Despite the limited genomic representation of predicted bacteria, the functional predictions were comparable to those of previous studies (Wang et al., 2019). At the KEGG category level abundances, as expected, most of the top categories shared a similar pattern of distribution (bacterial community in the bottom seawater was more abundant). Considering the enriched carbohydrate metabolism, as well as global and overview maps and amino acid metabolism, it was predicted that the functional capacity of bacterial communities is potentially more concentrated on nutrient metabolism in these highly productive ecosystems (Steen et al., 2008). However, a more comprehensive investigation and a detailed functional classification are required in biogeochemical cycling across the surface to bottom gradients in the coastal ocean. This gradient of predicted functions appears to be due to physiological adaptation (Thottathil et al., 2008) or carbohydrate or other substrate limitation with increasing depth. Additionally, uncovering the specific mechanisms of their ecological function is limited by only one period of sampling. To unveil more details, further study is urgently warranted to integrate time-series data and a more complete environmental profile.

## Conclusion

The study demonstrated clear spatial patterns in bacterial community composition, geographic distribution, underlying mechanism, co-occurrence and functional characteristics in marine waters from Bohai Sea to northern Yellow Sea. The differentiation of ecological niches, together with dispersal limitations and interspecies interaction relationships, collectively influences the spatial distribution patterns of bacterial communities. At the functional level, there is a more pronounced vertical distribution of the functional potential of bacterial communities, which could be explained by the stratification of the water column as well as the vertical distribution of multiple physicochemical factors, such as temperature, salinity, light and nutrients. Furthermore, we provided evidence that species abundance characteristics and turnover frequencies within bacterial communities were uncoupled from the functional characteristics of the communities. This may be due to the existence of a high degree of functional redundancy characteristic of bacterial communities, which is crucial for the functional stability of marine ecosystems.

## Data availability statement

The data presented in this study are deposited in the NCBI repository, accession number PRJNA917265.

## Author contributions

TN: conceptualization, methodology, formal analysis, investigation, resources, project administration, data curation, writing – original draft, writing – review and editing, visualization, software, and project administration. JL: conceptualization, methodology, validation, writing – review and editing, and supervision. YX, JC, and LQ: software and writing – review and editing. ZL and YY: writing – review and editing. All authors contributed to the article and approved the submitted version.

## Funding

This work was funded by the National Natural Science Foundation of China (No. 41666005) and the Natural Sciences Foundation of Guangxi (2018GXNSFAA281328).

## Conflict of interest

The authors declare that the research was conducted in the absence of any commercial or financial relationships that could be construed as a potential conflict of interest.

## Publisher’s note

All claims expressed in this article are solely those of the authors and do not necessarily represent those of their affiliated organizations, or those of the publisher, the editors and the reviewers. Any product that may be evaluated in this article, or claim that may be made by its manufacturer, is not guaranteed or endorsed by the publisher.

## Footnotes

^1^ http://www.r-project.org/

^2^ https://github.com/picrust/picrust2
