# Supplementary material for: Bacterial taxonomic and functional profiles from Bohai Sea to northern Yellow Sea
Source: Front Microbiol. 2023 Feb 23;14:1139950. doi: 10.3389/fmicb.2023.1139950 (PMC9995391; doi:10.3389/fmicb.2023.1139950)
Supplement: Supplementary file 1 [file Data_Sheet_1.docx]

Supplementary Material

Bacterial taxonomic and functional profiles from Bohai Sea to northern Yellow Sea

**Tianyi Niu, Yongqian Xu, Jinni Chen,** **Liangyun Qin, Zhicong Li, Yating Yang, Jiayuan Liang***

*** Correspondence:** Jiayuan Liang: jyliang@gxu.edu.cn

# Supplementary Figures and Tables

## Supplementary Figures


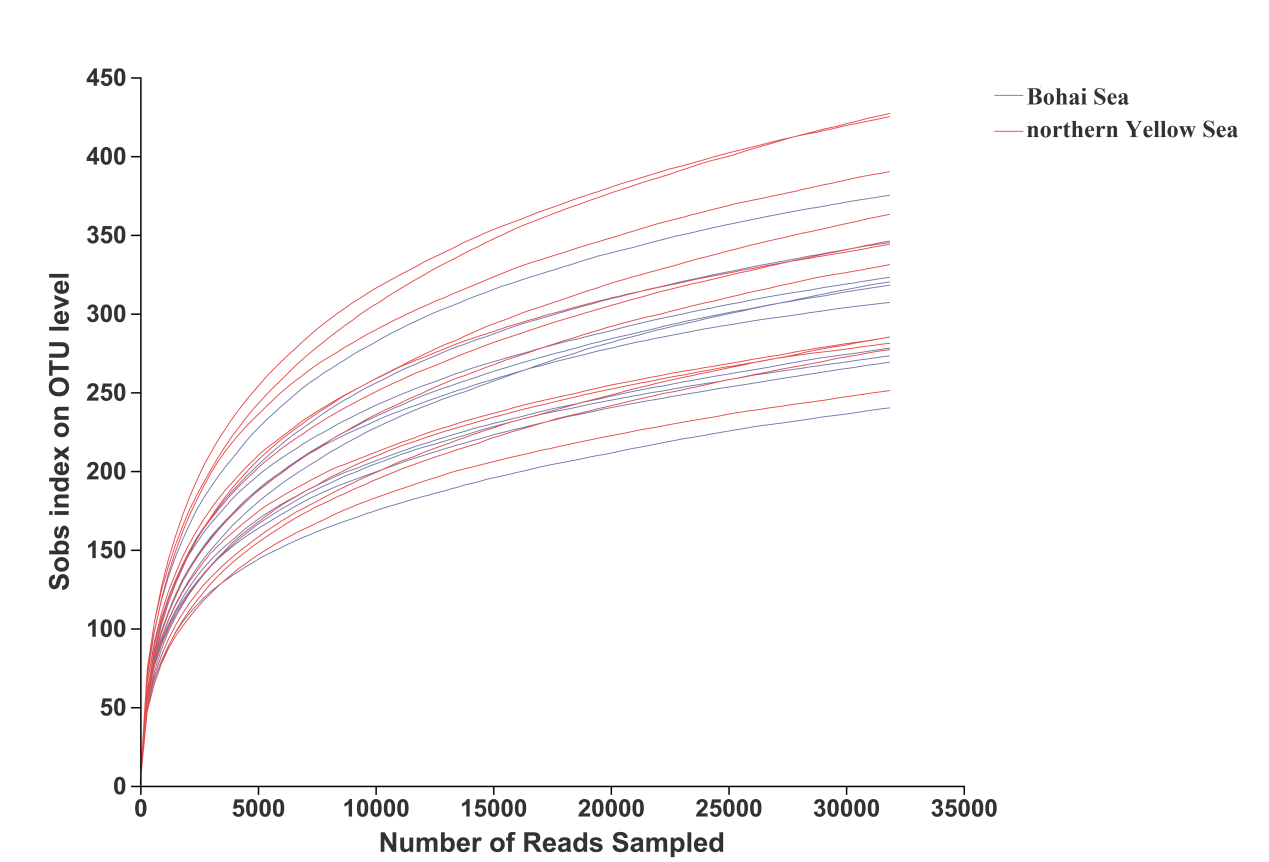


**Supplementary Figure 1.** Rarefaction curve of Sobs index based on 16S rRNA sequence.


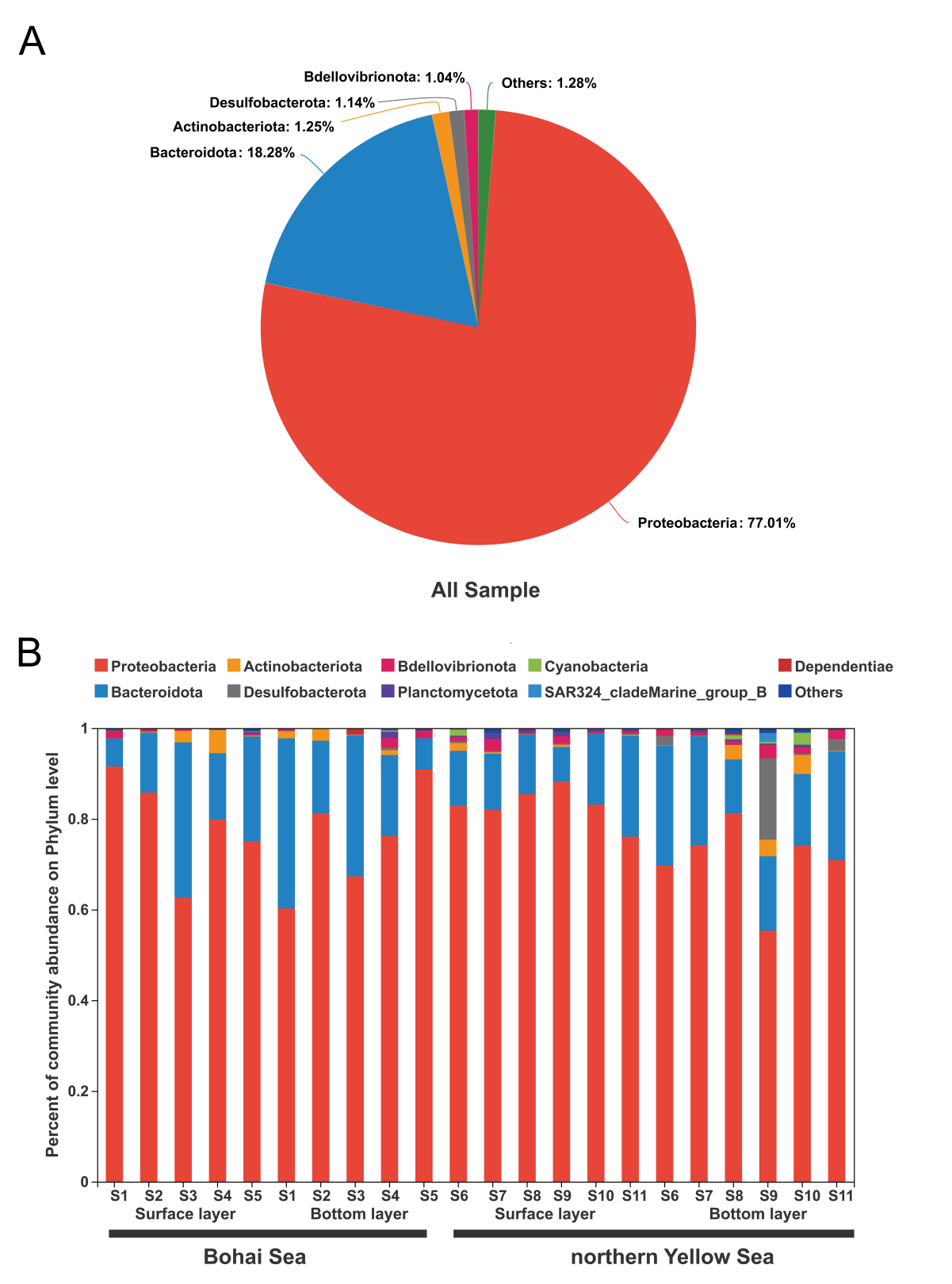


**Supplementary Figure 2.** Phylum-level comparison of bacterial community structure among all samples **(A)** and single samples **(B)**.


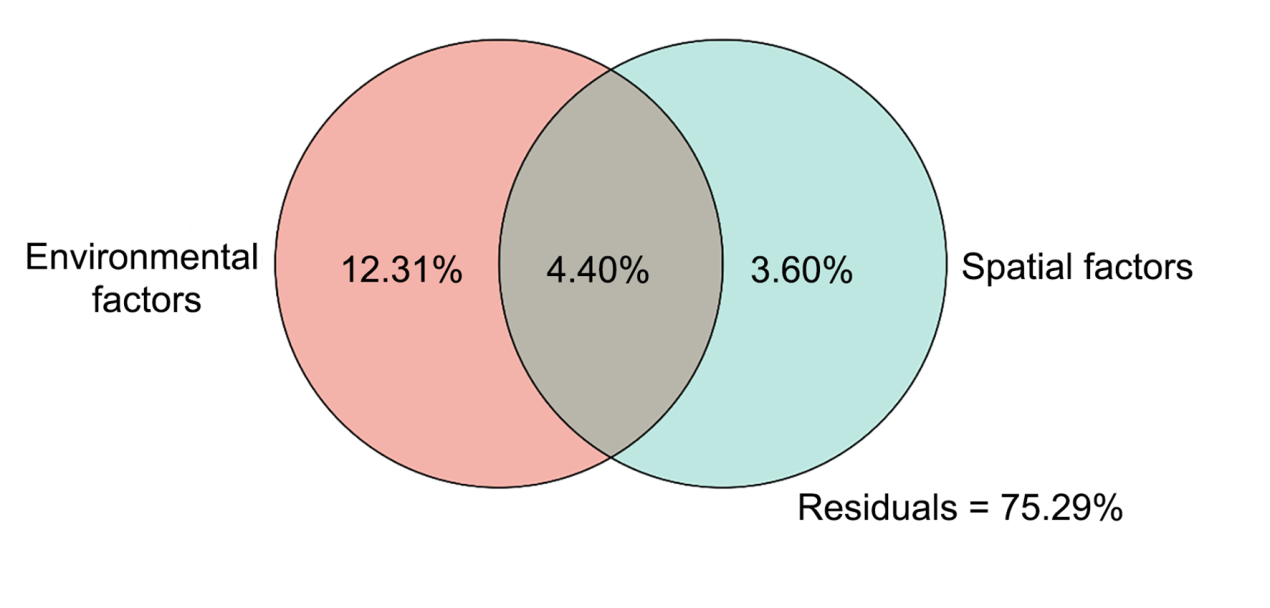


**Supplementary Figure 3.** Variation partitioning analysis (VPA) of the relative contributions of distance and environmental variables on variation in bacterial β-diversity.


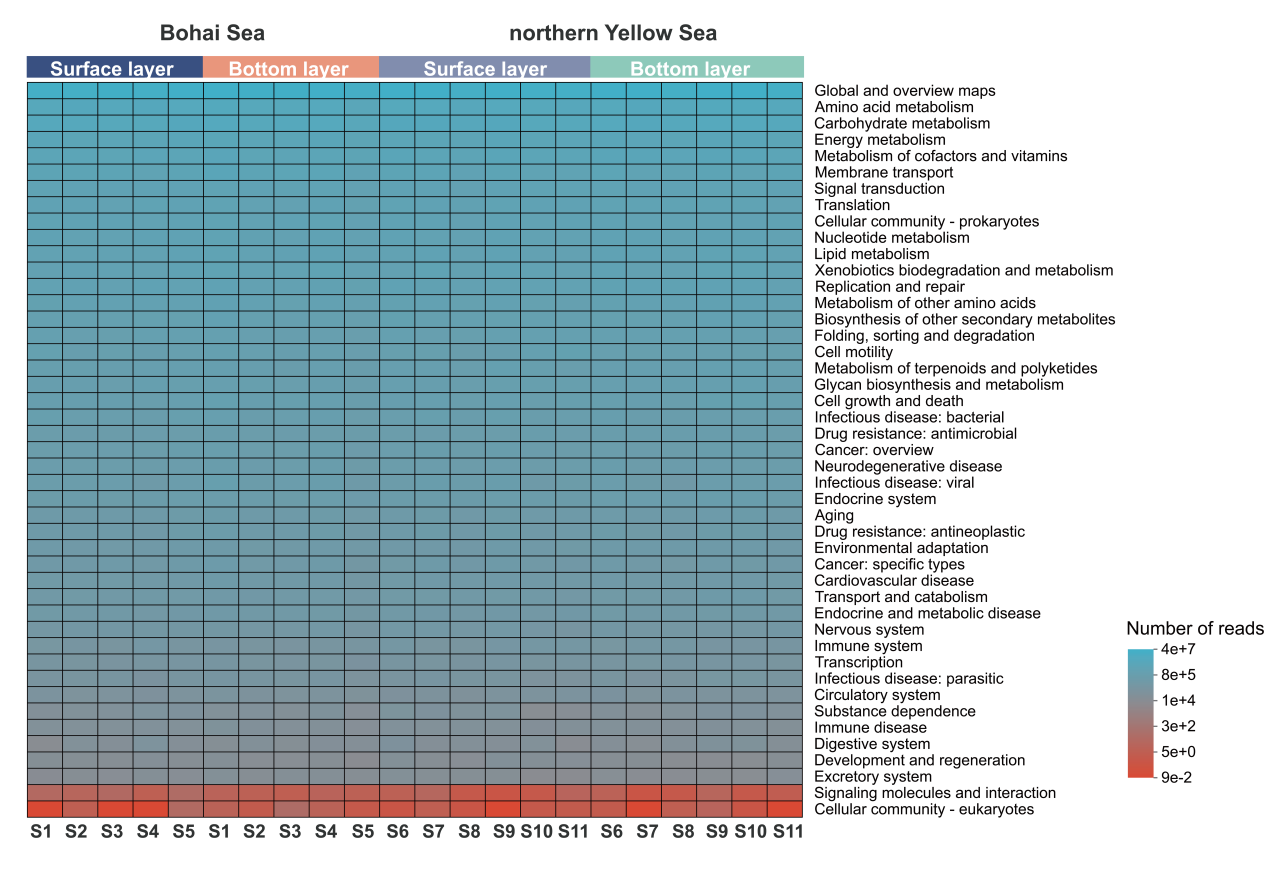


**Supplementary Figure 4.** Functional characteristics of bacterial communities on KEGG pathway 2. Heatmap shows the abundance in each function by a color gradient.

## Supplementary Tables

**Supplementary Table 1** Environmental parameters of sampling sites in this study.

| Sampling site | Groups | T (℃) | Salinity | PH | TP  (mg/l) | NH_4_^+^-N  (mg/l) | NO_2_^-^-N  (mg/l) | NO_3_^-^-N  (mg/l) | SSC  (g/l) | TOC  (mg/kg) | DO  (mg/l) |
| --- | --- | --- | --- | --- | --- | --- | --- | --- | --- | --- | --- |
| S1 | Surface layer | 13.660 | 28.600 | 8.380 | 12.510 | 0.487 | 0.255 | 0.775 | 0.070 | 0.990 | 7.685 |
| S2 |  | 14.990 | 29.300 | 8.350 | 7.360 | 0.346 | 0.201 | 0.114 | 0.150 | 1.636 | 6.587 |
| S3 |  | 12.690 | 26.500 | 8.310 | 9.770 | 0.211 | 0.294 | 0.800 | 0.105 | 1.020 | 8.025 |
| S4 |  | 15.640 | 25.700 | 8.430 | 8.940 | 0.181 | 0.515 | 0.762 | 0.106 | 0.987 | 7.666 |
| S5 |  | 14.320 | 28.300 | 8.230 | 3.350 | 0.184 | 0.028 | 0.742 | 0.245 | 2.254 | 7.654 |
| S1 | Bottom layer | 14.200 | 33.500 | 8.210 | 5.030 | 0.165 | 0.063 | 0.663 | 0.256 | 1.350 | 6.214 |
| S2 |  | 16.350 | 32.600 | 8.400 | 7.850 | 0.069 | 0.156 | 0.713 | 0.460 | 1.587 | 5.324 |
| S3 |  | 16.550 | 31.200 | 8.010 | 4.990 | 0.197 | 0.132 | 0.111 | 0.358 | 1.654 | 7.002 |
| S4 |  | 14.620 | 32.700 | 8.180 | 4.170 | 0.087 | 0.001 | 0.077 | 0.258 | 2.054 | 6.335 |
| S5 |  | 14.340 | 32.600 | 8.400 | 3.200 | 0.076 | 0.003 | 0.665 | 0.354 | 3.258 | 5.998 |
| S6 | Surface layer | 14.660 | 27.500 | 8.110 | 7.111 | 0.274 | 0.149 | 0.925 | 0.076 | 0.987 | 6.353 |
| S7 |  | 13.490 | 26.700 | 8.730 | 4.830 | 0.204 | 0.020 | 0.880 | 0.058 | 1.023 | 7.542 |
| S8 |  | 13.520 | 27.400 | 8.210 | 6.560 | 0.199 | 0.041 | 1.281 | 0.065 | 1.265 | 8.325 |
| S9 |  | 14.140 | 32.000 | 8.160 | 3.260 | 0.177 | 0.059 | 0.608 | 0.058 | 1.336 | 7.998 |
| S10 |  | 13.650 | 28.900 | 8.000 | 12.820 | 0.537 | 0.117 | 0.685 | 0.076 | 1.698 | 9.254 |
| S11 |  | 15.680 | 36.300 | 8.070 | 41.500 | 0.042 | 0.039 | 0.623 | 0.199 | 2.465 | 6.257 |
| S6 | Bottom layer | 13.250 | 32.500 | 8.160 | 3.890 | 0.298 | 0.015 | 0.097 | 0.135 | 0.690 | 6.358 |
| S7 |  | 15.320 | 33.700 | 8.160 | 4.260 | 0.085 | 0.399 | 0.291 | 0.354 | 0.998 | 6.254 |
| S8 |  | 15.260 | 30.500 | 8.460 | 1.960 | 0.314 | 0.050 | 0.034 | 0.066 | 1.275 | 7.365 |
| S9 |  | 15.200 | 34.500 | 8.020 | 2.600 | 0.071 | 0.010 | 0.110 | 0.177 | 2.225 | 7.665 |
| S10 |  | 15.060 | 34.600 | 7.990 | 1.650 | 0.066 | 0.008 | 0.102 | 0.123 | 1.998 | 7.354 |
| S11 |  | 16.020 | 35.400 | 8.110 | 21.250 | 0.061 | 0.035 | 0.508 | 0.321 | 3.256 | 6.923 |

| Areas | Sampling site | Groups | Sobs | Shannon | Simpson | ACE | Chao 1 | Coverage |
| --- | --- | --- | --- | --- | --- | --- | --- | --- |
| Bohai Sea | S1 | Surface layer | 240 | 2.910411 | 0.144503 | 361.9461 | 290.8333 | 0.998086 |
|  | S2 |  | 278 | 3.052886 | 0.164287 | 353.5174 | 370.0370 | 0.997772 |
|  | S3 |  | 269 | 3.187142 | 0.105716 | 333.5208 | 343.2857 | 0.997961 |
|  | S4 |  | 273 | 3.390289 | 0.064156 | 328.8242 | 332.0323 | 0.998086 |
|  | S5 |  | 323 | 3.576673 | 0.067409 | 387.0601 | 386.4054 | 0.997835 |
|  | S1 | Bottom layer | 375 | 3.533026 | 0.099591 | 433.7945 | 436.3864 | 0.997678 |
|  | S2 |  | 318 | 3.460768 | 0.067328 | 390.9612 | 399.6176 | 0.997647 |
|  | S3 |  | 345 | 2.922745 | 0.191806 | 405.0327 | 422.0833 | 0.997647 |
|  | S4 |  | 320 | 3.454818 | 0.063782 | 402.0676 | 407.1500 | 0.997365 |
|  | S5 |  | 307 | 3.039042 | 0.138284 | 355.1902 | 345.4419 | 0.998180 |
| northern Yellow Sea | S6 | Surface layer | 281 | 3.193247 | 0.097483 | 333.2335 | 329.4872 | 0.998055 |
|  | S7 |  | 251 | 3.242077 | 0.074342 | 316.5160 | 316.0323 | 0.997992 |
|  | S8 |  | 277 | 3.022971 | 0.099269 | 418.6702 | 385.0000 | 0.997459 |
|  | S9 |  | 427 | 3.725702 | 0.069075 | 530.0886 | 551.5918 | 0.996518 |
|  | S10 |  | 285 | 2.942206 | 0.154593 | 377.5305 | 370.2632 | 0.997459 |
|  | S11 |  | 363 | 3.262806 | 0.101583 | 467.4068 | 478.9268 | 0.996925 |
|  | S6 | Bottom layer | 285 | 3.308472 | 0.085689 | 355.6430 | 397.9545 | 0.997772 |
|  | S7 |  | 331 | 3.535541 | 0.058737 | 425.4320 | 431.1250 | 0.997176 |
|  | S8 |  | 346 | 3.644633 | 0.056268 | 446.1169 | 445.8780 | 0.997145 |
|  | S9 |  | 344 | 3.596047 | 0.062841 | 408.5591 | 447.5517 | 0.997553 |
|  | S10 |  | 390 | 3.873989 | 0.040129 | 479.8237 | 483.1395 | 0.997176 |
|  | S11 |  | 425 | 3.879574 | 0.042865 | 514.0562 | 524.0638 | 0.996957 |

**Supplementary Table 2** α-diversity characteristics of the bacterial community in the Bohai and northern Yellow Seas water.
